# A rapid fluorometric assay for the S-malonyltransacylase FabD and other sulfhydryl utilizing enzymes

**DOI:** 10.14440/jbm.2016.144

**Published:** 2016-09-07

**Authors:** Aaron M. Marcella, W. Adam Barb

**Affiliations:** Roy J. Carver Department of Biochemistry, Biophysics and Molecular Biology, Molecular Biology Building, Room 4210, 2437 Pammel Drive, Iowa State University, Ames, IA 50011, USA

**Keywords:** FabD, FabH, CPM, high throughput, enzyme kinetics

## Abstract

The development of biorenewable chemicals will support green chemistry initiatives and supplement the catalog of starting materials available to the chemical industry. Bacterial fatty acid biosynthesis is being pursued as a source of protein catalysts to synthesize novel reduced carbon molecules in fermentation systems. The availability of methods to measure enzyme catalysis for native and engineered enzymes from this pathway remains a bottleneck because a simple quantitative enzyme assay for numerous enzymes does not exist. Here we present two variations of a fluorescence assay that is readily extendable to high-throughput screening and is appropriate for thiol consuming and generating enzymes including the *E. coli* enzymes malonyl-coenzyme A transacylase (FabD) and keto-acylsynthase III (FabH). We measured *K*_M_ values of 60 ± 20 µM (acetyl-CoA) and 20 ± 10 µM (malonyl-ACP) and a *k*_cat_ of 7.4–9.0 s^-1^ with FabH. Assays of FabD included a precipitation step to remove the thiol-containing substrate holo-ACP from the reaction product coenzyme-A to estimate reaction rates. Analysis of initial velocity measurements revealed *K*_M_ values of 60 ± 20 µM (malonyl-CoA) and 40 ± 10 µM (holo-ACP) and a *k*_cat_ of 2100–2600 s^-1^ for the FabD enzyme. Our data show similar results when compared to existing radioactive and continuous coupled assays in terms of sensitivity while providing the benefit of simplicity, scalability and repeatability. Fluorescence detection also eliminates the need for radioactive substrates traditionally used to assay these enzymes.

## INTRODUCTION

Fatty acid biosynthesis produces reduced hydrocarbon chains and is a target of engineering efforts to develop novel routes to biorenewable chemicals [1]. Prokaryotic fatty acid biosynthesis is well characterized and a popular target for drop-in enzyme replacements due to implementation as a one-polypeptide/one-activity system unlike eukaryotic synthases that are expressed as a single fused polypeptide and likely more challenging to engineer due to inter-subunit contact [2-4]. Biorenewable chemicals are increasingly being produced in fermentative systems (ethanol, arachidonic acid, docosahexaenoic acid, short and medium chain fatty acids and others [5-7]) and future needs will likely be met by recombinant microbes that express engineered enzymes and secrete novel materials.

Fatty acid biosynthesis builds a carbon chain on acyl carrier protein (ACP) two carbon units at a time. Acetyl-coenzyme A (acetyl-CoA) donates the two acetate carbons to prime the chain and the first reaction between the acetyl:enzyme intermediate with malonyl-ACP generates acetoacetyl-ACP, carbon dioxide and CoA-SH. Acetoacetyl-ACP is reduced to butyryl-ACP and extended again using malonyl-ACP as the source of material. This extension/reduction cycle continues until the chain reaches the prescribed length and is cleaved from the acyl carrier protein or transferred directly to a lipid molecule.

**Figure 1:**
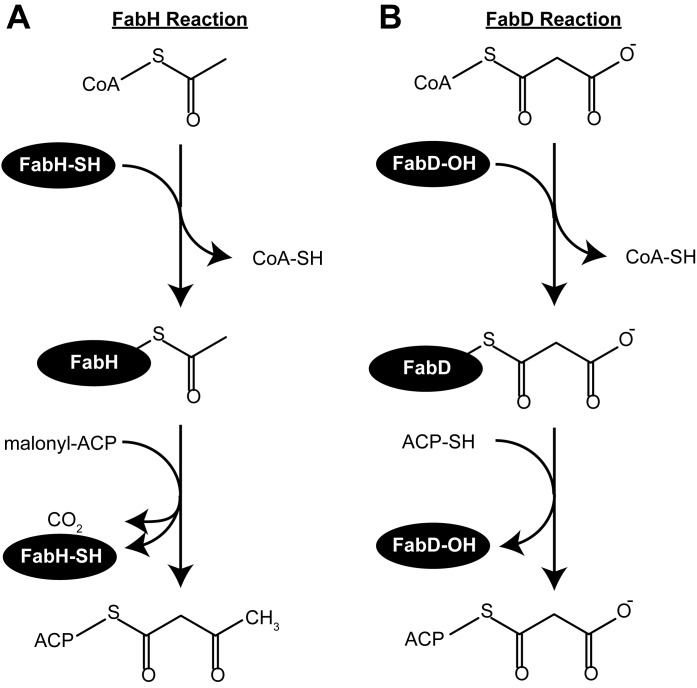
**Reactions catalyzed by FabH and FabD**. **A**. FabH creates an acetyl-enzyme covalent intermediate at C112, releasing CoA-SH. Nucleophilic attack of the acetate by the decarboxylated malonyl-ACP forms acetoacetyl-ACP. **B**. FabD catalysis begins with the generation of a covalent intermediate at S92. This releases CoA-SH and allows for holo-ACP to attack the malonate intermediate on FabD creating malonyl-ACP.

Enzymes that generate carbon-carbon bonds, including the keto-acylsynthases (KAS), and enzymes that generate primers and extender units utilized by the keto-acylsynthases are of particular interest for biorenewable production. The β-ketoacyl-ACP synthase III (FabH) catalyzes the first priming reaction in *E. coli* fatty acid biosynthesis as shown in ****Figure 1****. The structure and activity of FabH is well characterized [8, 9]. FabH recognizes either malonyl-CoA or malonyl-ACP as an extender unit and this versatility marks this enzyme as an engineering target as opposed to the potentially more limited KASI or KASII that require ACP substrates [10]. Designing FabH to recognize acetyl-CoA derivatives would impart chemical diversity at the ω end of fatty acids.

Diversification of fatty acid biosynthesis pathways may also be achieved by incorporating alternative extender units into the growing chain and would affect its entire length. In fatty acid biosynthesis, the malonyl-CoA:ACP transacylase (FabD) generates the extender units by transferring the malonyl moiety from malonyl-CoA to holo-ACP, forming the free thiol form of CoA (CoA-SH) and malonyl-ACP as shown in **Figure 1**. The result is no net change in free thiol concentration and this assay cannot be monitored directly with thiol reactive reagents unless the holo-ACP and CoA-SH can be separated. Diversification of substrate recognition by FabD would be required to modify the extender units incorporated during chain elongation.

In vitro strategies to screen and characterize designed enzymes require an enzyme assay that is applicable to many of the diverse fatty acid biosynthesis enzymes, including FabD and FabH, and capable of high throughput to thoroughly analyze numerous target substrates and enzyme variants. Sulfhydryl containing compounds including CoA-SH and holo-acyl carrier protein (holo-ACP) are utilized during biosynthesis and represent one common feature of many fatty acid biosynthesis enzymes that is detectable with high sensitivity in high throughput assays [11, 12]. To date, most published approaches to measuring reactions catalyzed by the enzymes of fatty acid biosynthesis utilize radioactive isotopes. While sensitive, these approaches are neither high throughput nor easily scalable [13-16]. Linked assays are also reported, but are neither easily adaptable to other enzymes in the pathway and [17], in our laboratory, were limited by contaminants in the commercial enzyme preps (data not shown).

**Figure 2:**
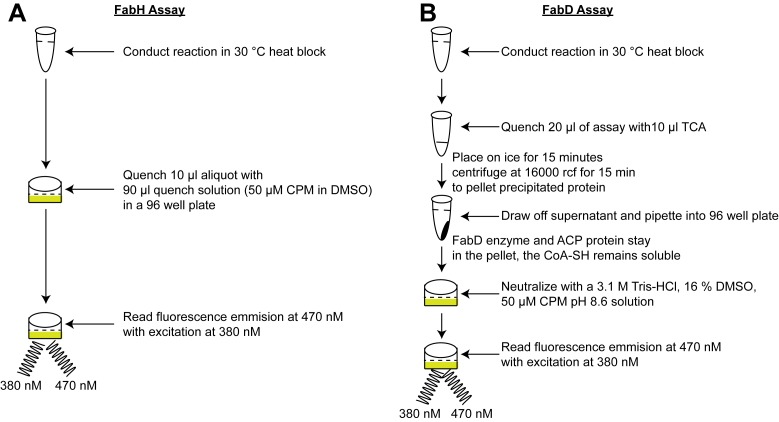
**Flow diagram for the FabH (A) and FabD (B) assays**. Both reactions utilize the reaction of CoA-SH with CPM to quantify product formation in the final step. The FabD assay differs because holo-ACP must be removed.

Here we describe a fluorescence-based strategy that can function with numerous enzymes in the FAS pathway including FabH and FabD at high sensitivity and is applicable to high throughput screening. Common implementations of thiol reactive reagents require a net thiol change which is problematic for detecting FabD activity through a liberated thiol as noted above [11]. We incorporated a facile step to separate CoA-SH from holo-ACP with reproducible and quantitative yields (**Fig. 2**). Free thiols, generated from the reaction cycle, are detected with 7-diethylamino-3-(4'-maleimidylphenyl)-4-methylcoumarin (CPM) [18]. This detection strategy is applicable to multiple enzymes and has proven to be reliable and sensitive [11, 12].

## MATERIALS AND METHODS

All materials were purchased from Sigma Aldrich unless otherwise noted.

### FabD preparation

The open reading frame for the *E. coli* FabD gene was cloned into pET28b using the *Nco*I and *Eco*RI sites. This cloning strategy adds only an extra glycine at the N-terminal of the enzyme and does not utilize the N or C-terminal poly-histidine tags available in the vector sequence. The EcFabD pET28b vector was transformed into *E. coli* BL21* DE3 cells and a single colony was selected then grown overnight in 5 ml Luria-Bertani (LB) medium supplemented with 50 μg/ml kanamycin. This culture was then added to 1 l LB with 50 μg/ml kanamycin and grown to mid log phase (OD600~0.5) at 37°C in a shaking incubator (Thermo Scientific MaxQ 4000). At this time fresh Isopropyl β-D-1-thiogalactopyranoside was added to a final concentration of 0.5 mM and incubated with shaking for approximately 18 h at 18°C. Cells were then pelleted by centrifugation and resuspended in 30 ml lysis buffer (25 mM MOPS, 1 mM EDTA, 1 mM BME, pH 7.1). The cell resuspension was lysed in an Emulsiflex C5 homogenizer (Avestin) 5 times at 12000 psi and the lysed cell debris pelleted at 24500 rcf in a Fisher Scientific Sorvall Legend XTR centrifuge for one hour. The clarified lysate was applied to a 110 ml Q-sepharose column (GE life sciences) and purified using two anion exchange buffers, A (lysis buffer) and B, lysis buffer supplemented with 1 M potassium chloride. The column was washed with 0.5 column volume (CV) of buffer A followed by a linear gradient from 5%–50% buffer B over one column volume. The column was washed with 0.2 CV of 100% buffer B and finally 1.2 CV of 100% buffer A. Fractions were collected from the start of the gradient until the end of the run and the FabD enzyme elutes at ~30% B.

Following anion exchange chromatography the fractions containing FabD were concentrated using a 10 kDa cutoff amicon ultracentrifugation filter unit (EMD Millipore) and applied to a Superdex S200 column (GE) equilibrated with 25 mM MOPS, 100 mM sodium chloride, 1 mM DTT, pH 7.1. Fractions containing FabD were concentrated as above and stored in 50% glycerol at −80°C. FabD was quantified using the absorbance at 280 nm and an extinction coefficient of 32680 M^-1^cm^-1^.

### FabH preparation

FabH was purified using anion exchange chromatography as described previously [11]. The two buffers used were buffer A: 20 mM phosphate, 1 mM EDTA, 5 mM BME pH 7.0 and buffer B which is buffer A with 1 M potassium chloride. For FabH a linear gradient of buffer B was applied to the anion exchange column in the same manner as FabD. Following anion exchange chromatography the fractions containing FabH were pooled and applied to a blue sepharose column (GE) and a linear gradient of buffer B was used in the same way as the anion exchange step. Fractions containing FabH from the blue sepharose column were pooled and concentrated with a 10 kDa amicon filter and applied to an S200 column (GE). The S200 column was equilibrated and eluted with a buffer containing 20 mM phosphate, 50 mM KCl, 1 mM DTT, pH 7.0. Fractions containing FabH were pooled, concentrated as above and stored at 4°C. The FabH extinction coefficient used for quantification was 25690 M^-1^ cm^-1^.

### Holo-ACP preparation

Expression and purification of holo-ACP has been described previously [11]. Briefly, the acyl-carrier-protein and holo-(acyl-carrier-protein) synthase genes were cloned into a pETDUET vector (EMD-Millipore). Both proteins were expressed simultaneously following the same steps as FabD expression (above). After lysing and centrifuging the cells the clarified lysate was applied to an 8 ml nickel column (Qiagen) and an imidazole gradient was run from 0–500 mM imidazole in buffers containing 20 mM Tris-HCl, 500 mM sodium chloride, pH 8.1. Fractions containing holo-ACP were diluted to reduce sodium chloride to 100 mM and loaded onto a 110 ml Q-Sepharose column. A linear gradient from 200–600 mM potassium chloride was applied to the column and the holo-ACP eluted around 500 mM potassium chloride. Fractions containing ACP were concentrated and exchanged into a buffer containing 25 mM MOPS, 100 mM sodium chloride, 1 mM DTT, pH 7.1 using a Superdex S75 column (GE). Fractions containing holo-ACP were concentrated using a 3 kDa amicon filter unit and stored at −80°C in 10% glycerol. The ACP extinction coefficient used for quantification was 1490 M^-1^cm^-1^.

### Malonyl-ACP preparation

Malonyl-ACP for the FabH assays was prepared as described previously [11]. Briefly, malonyl-CoA and holo-ACP were combined at a 25:1 (mole:mole) ratio with 5 µM FabD in 5 ml of 25 mM MOPS, 100 mM sodium chloride, and 1 mM DTT, pH 7.0. After 5 h at 0°C the reaction was applied to a 1 ml nickel column. The column was washed 2 × (5 ml each wash) with a buffer containing 20 mM Tris-HCl, 500 mM sodium chloride, pH 8.1, and eluted with 5 × 1 ml aliquots of the above buffer supplemented with 250 mM imidazole. Fractions containing Malonyl-ACP were pooled concentrated and exchanged into a buffer containing 25 mM MOPS, 100 mM sodium chloride, pH 7.1, using an Amicon-Ultra 15 ml 3 kDa MWCO filter unit and stored at −80°C following addition of glycerol to 10% v/v. The proportion of malonyl-ACP in the total ACP pool was estimated at 40% using MALDI-TOF analysis; major contaminants include acetyl-ACP and holo-ACP (data not shown).

### FabD assay

Below is a description of the materials required for the FabD-catalyzed reaction (**Fig. 2B**).

#### Reagents

• Wash buffer (25 mM MOPS, 100 mM sodium chloride, pH 7.1)
• Assay buffer (25 mM MOPS, 100 mM sodium chloride, 1 mg/ml BSA, pH 7.1)
• FabD Quench solution 1 (150% w/v TCA)
• FabD Quench solution 2 (95% w/v TCA)
• 1 mM CoA-SH
• 100 µM CoA-SH
• 3 mM holo-ACP
• 10 mM malonyl-CoA
• 1 nM FabD
• 3.75 M Tris-HCl (pH 8.6)
• 100% DMSO
• 10 mM CPM in DMSO
• Neutralization solution (3.125 M Tris-HCl, 16.6% DMSO, 50 µM CPM, pH 8.6)

Below is a description of the steps performed to measure the FabD-catalyzed reaction.

1. Exchange FabD and holo-ACP into 25 mM MOPS, 100 mM sodium chloride, pH 7.1 buffer to reduce the concentration of DTT by about 1000 fold.
2. Conduct assays in 200 or 800 µl volumes depending on the total amount of ACP or malonyl-CoA in the assay, the higher reaction volume is needed to increase sensitivity for assays with low substrate concentrations.
3. An example 200 µl reaction is prepared with 4.7 µl 3 mM holo-ACP (70 µM final), 20 µl 1 nM FabD enzyme (100 pM), with assays being brought to final volume (196 µl) with 171.3 µl assay buffer in a 1.5 ml tube.
4. Place reactions in a 30°C heat block to warm for 1 min. Start assays with 4 µl of 10 mM malonyl-CoA (200 µM final).
5. Aliquots (20 µl) are taken at intervals of 10 s for 1 min. Aliquots are immediately placed into FabD quench solution 1 then incubated on ice for five min (for 800 µl reactions, quench 80 µl into 40 µl FabD quench solution 2 and place on ice). Quenched reactions are centrifuged at 16000 × g in a microcentrifuge at 4°C for 15 min to pellet the protein.
6. Draw off the supernatant (20 µl) and pipette into a 96 well dark sided plate with clear bottom (Thermo Fisher). Neutralization solution (50 µl) is added to the plates and mixed thoroughly. It is critical to use enough Tris-HCl to ensure proper buffering of the reaction solution and complete reaction with the CPM reagent. This can be verified by applying ~1 µl of neutralized reaction to pH paper.
7. Read plates in a plate reader (Tecan Safire) with an excitation wavelength of 380 nm and emission at 470 nm. The slit width was 5 nm for both excitation and emission; data were collected with 10 flashes per well and an integration time of 401397645907s. Rates of product accumulation are fitted with a line over the linear portion of the assays using MS-Excel. Fits of the Michaelis-Menten equation were performed using DynaFit (www.biokin.com/dynafit/).
8. Make a standard curve using assay buffer and CoA-SH. For the 200 µl volume assays, mix 20 µl CoA-SH solution in reaction buffer with 10 µl FabD quench solution 1, place on ice for 15 min, and then centrifuge at 16000 × g and 4°C. Neutralize 20 µl of the quenched standard curve solution with 50 µl neutralization solution. This yields a final volume for fluorescence analysis of 70 µl, the same as for the assay. Concentrations of CoA-SH in the 20 µl samples are as follows: 0 µM, 2.625 µM, 5.25 µM, 10.5 µM, 26.25 µM, and 52.5 µM. This standard curve is used to calculate the concentration of CoA-SH produced in the assays.

### FabH assay

FabH assays were conducted similarly to FabD, but the precipitation step is not required because only one thiol is generated (CoA-SH) and none are consumed.

#### Reagents

• Wash buffer (20 mM phosphate, 50 mM potassium chloride, pH 7.0)
• Assay buffer (20 mM phosphate, 50 mM potassium chloride, 1 mg/ml BSA, pH 7.0)
• FabH Quench solution (50 µM CPM in DMSO)
• 1 mM acetyl-CoA
• 2 mM malonyl-ACP
• 100 nM FabH
  1. Exchange FabH into wash buffer to reduce the DTT concentration at least 1000 ×.
  2. Perform assays at 30°C in 100 µl volumes with 7.5 µl, 1 mM mM acetyl-CoA (75 µM), 3.5 µl, 2 mM malonyl-ACP (70 µM), and 79 µl assay buffer.
  3. Start reactions with 10 µl of 100 nM FabH (10 nM). Aliquots were taken at 1, 2, 4, 6, 8 and 10 min. At each time point 10 µl of the assay was removed from the reaction vessel then immediately quenched into 90 µl quench solution in a 96 well plate. The samples were read in a Tecan Safire plate reader with excitation wavelength of 380 nm and emission at 470 nm.

## RESULTS AND DISCUSSION

**Figure 3:**
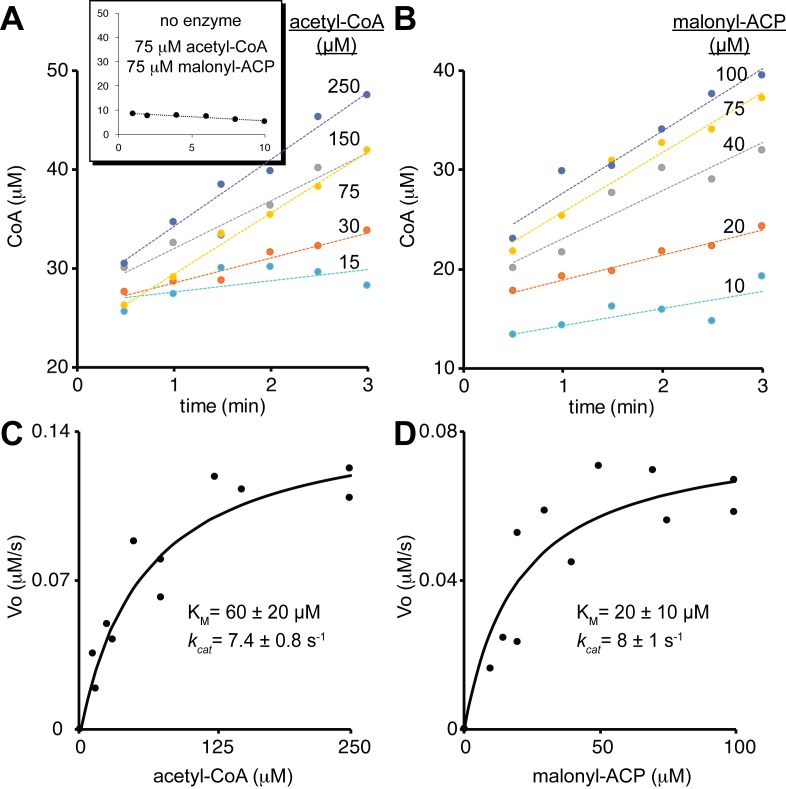
**Saturation kinetics experiments for *E. coli* FabH**. **A**. Progress curves for FabH reactions with varying concentrations of acetyl-CoA and 80 µM malonyl-ACP with 20 nM FabH and 10 nM FabH, respectively. Inset: no enzyme control. **B**. Progress curves of FabH reactions with varying concentrations of malonyl-ACP and 200 µM acetyl-CoA. **C**. A plot of reaction rates versus acetyl-CoA concentration. **D**. A plot of reaction rates versus malonyl-ACP concentration. Dashed lines represent a linear fit to product accumulation data and solid lines represent a fit of the Michaelis-Menten equation.

We demonstrated the measurement of FabH activity using the described method and found an increase in fluorescence over time. Single representative experiments are shown in **Figure 3A** and **3B**. Reactions without enzyme repeatedly showed flat profiles (**3A** inset). Each experiment revealed product accumulation data from 6 separate reactions resulting in 36 individual measurements that required one person about 45 min to complete from start to finish.

To obtain estimates of Michaelis constants (*K*_M_) for each substrate as well as catalytic rates (*k*_cat_), one substrate was held at a concentration ~4 fold higher than its *K*_M_ while the other substrate was varied. We fit the Michaelis-Menten equation to plots of the initial velocities versus the varied substrate concentration to estimate *K*_M_ (acetyl CoA 60 ± 20 M; malonyl-ACP 20 ± 10 M) and *k*_cat_ (7.4 ± 0.8 s^-1^ and 8.0 ± 1.0 s^-1^, respectively). Data in **Figure 3C** and **3D** include the data from **Figure 3A** and **3B**, respectively, and include additional data from independent experiments collected on different days to demonstrate the reproducibility and variability of data collected using this method. These data are shown in the raw form and individual experiments were not scaled for comparison which clearly demonstrates the high degree of repeatability and stability of the assay. **Table 1** shows the resulting values measured using this method are comparable to previously measured values.

The assay of FabH activity proved sensitive due to the generation of a free thiol (in the form of CoA-SH) during the reaction cycle. Assays of FabD activity could not use the same experimental approach because though a free thiol is generated (CoA-SH) during the reaction, one is consumed (holo-ACP) resulting in no net change in measurable CPM fluorescence (**Fig. 1**). We developed a method that selectively precipitated the substrate holo-ACP from the reaction mixture, leaving the product CoA-SH in solution. This solution could then be probed for free thiol content using the CPM reagent.

**Figure 4:**
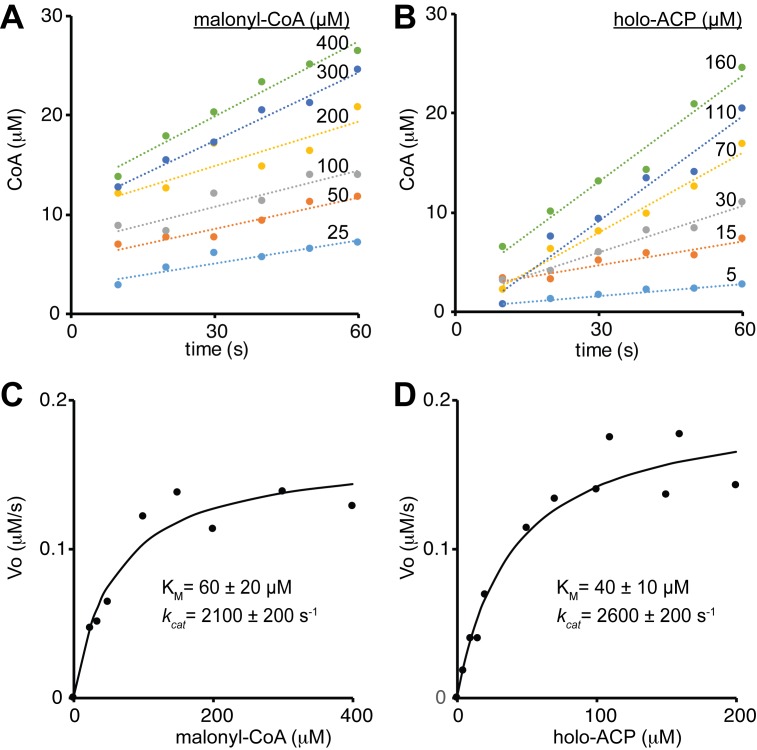
**Saturation kinetics experiments for *E. coli* FabD**. **A**. Progress curves for FabD reactions with varying concentrations of malonyl-CoA and 125 µM holo-ACP. **B**. Progress curves of FabD reactions with varying concentrations of holo-ACP and 250 µM acetyl-CoA. **C**. A plot of reaction rates versus malonyl-CoA concentration. **D**. A plot of reaction rates versus holo-ACP concentration. Dashed lines represent a linear fit to product accumulation data and solid lines represent a fit of the Michaelis-Menten equation.

The FabD activity assay likewise proved sensitive and repeatable. Initially, multiple assays were conducted to determine optimal enzyme and substrate concentrations for saturation kinetics experiments (data not shown). Once conditions were identified, product accumulation was measured as shown in **Figure 4A** and **4B**. Linearity over time is evident across all substrate concentrations assayed with only minor noise present at the lower substrate concentrations. Furthermore, observed reaction rates reached saturation at high substrate concentrations and permitted *K*_M_ and *k*_cat_ estimation by fitting the Michaelis-Menten equation as shown in **Figure 4C** and **4D**. Data in **Figure 4C** and **4D** show an overlay from three independent experiments collected on three different days. Similar to that observed for FabH, these data are shown in the raw form and individual experiments were not scaled for comparison which clearly demonstrates the high degree of repeatability and stability of the assay. Based on these analyses we estimated *K*_M_ values of 40 ± 10 µM for holo-ACP and 60 ± 20 µM for malonyl-CoA. Values for *k*_cat_, measured from each saturation curve, closely agreed at 2600 ± 300 s^-1^ and 2100 ± 200 s^-1^. The comparison of the values reported herein closely agree with previously reported values shown in **Table 2** [17, 19-21].

FabD assays required care to ensure complete precipitation of holo-ACP during the quench step. Multiple TCA concentrations were tested and it should be noted that at excessive TCA concentration, holo-ACP initially precipitated but was subsequently hydrolyzed into soluble peptides that led to higher than expected CPM fluorescence. This was evident by the appearance and subsequent rapid (min) disappearance of a white fluffy precipitate. ACP hydrolysis can be controlled by carefully tuning the TCA concentration for each different aliquot volume. For 20 µl aliquots, 10 µl 150% w/v TCA provided superior results but 80 µl aliquots required 40 µl 90% w/v TCA. It also proved important to check the final pH of the neutralized solution before fluorescence measurements. A pH greater than 8.0 is necessary to ensure complete and rapid reaction of the CPM with CoA-SH. It is important to note that CPM is light sensitive, and fluorescence should be measured within one hour from the time of neutralization. It should also be noted that the difference in CPM fluorescence observed in both **Figure 3A** and **3B** and **Figure 4A** and **4B** is likely due to the contribution of contaminating free thiol in the ACP preparation.

This study introduces a method for safe and repeatable assays of fatty acid biosynthesis enzymes that utilize sulfhydryls. This method functions with exceptional repeatability and stability. Reliability will prove beneficial for adaptation to high throughput screening and allow for rapid analysis of many enzymes, substrates and potentially inhibitors. Our approach utilizes commercially available reagents and functions without radioactive substrates or linking enzymes with linking substrates that can complicate analysis. Although sample handling in the assay is more involved than a continuous coupled assay, it is still straightforward and completed in a short amount of time.

Thiol quantitation using CPM lends additional benefits to the approach described here. These methods will be directly applicable to studies of other fatty acid synthesis enzymes, including the KASI and KASII enzymes, as well as polyketide synthases that liberate thiols in the form of CoA-SH and generate ACP-bound intermediates within the reaction cycle [22, 23]. Furthermore, the assay method is also applicable in screens of drug molecule libraries for novel antibiotics targeting FabD, FabH or other FAS enzymes [24].

**Table 1:** FabH kinetic data.

Reference	Organism	Mal-ACP K_M_ (μM)	Acetyl-CoA K_M_ (μM)	k_cat_1 (s^-1^)*	k_cat_ 2 (s^-1^)**
This study	*E. coli*	20 ± 10	60 ± 20	8.0 ± 1.0	7.4 ± 0.8
Heath *et al.* [13]	*E. coli*	5	40	n.r.	n.r.
Qiu *et al.* [25]	*S. aureus*	19 ± 7	373 ± 17	261 ± 34	431 ± 113
Khandekar *et al.* [26]	*S. pneumoniae*	18.6 ± 1.5	40.3 ± 2.3	n.r.	n.r.

*k_cat_1 measured with 75 µM malonyl-ACP and saturating acetyl-CoA; **k_cat_2 measured with 250 µM acetyl-CoA and saturating malonyl-ACP; n.r. = data not reported

**Table 2:** FabD kinetic data.

Reference	Organism	Holo-ACP K_M_ (μM)	Malonyl-CoA K_M_ (μM)	k_cat_1 (s^-1^)**	k_cat_ 2 (s^-1^)***
This study	*E. coli*	40 ± 10	60 ± 20	2600 ± 300	2100 ± 200
Molnos *et al.* [17]	*E. coli*	19	25	n.r.	n.r.
Joshi *et al.* [19]	*E. coli*	54	25	n.r.	n.r.
Szfranska *et al.* [20]	*S. coelicolor*	73	60	450	n.r.
Kremer *et al.* [21]	*M. tuberculosis*	14	50*	n.r.	n.r.

*Refit of published data with non-linear least squares fitting software (reported 12.6 µM); **k_cat_1 measured with 250 µM malonyl-CoA and saturating holo-ACP; ***k_cat_2 measured with 125 µM holo-ACP and saturating malonyl-CoA; n.r. = data not reported

## Abbreviations used

FAS: fatty acid synthase
FabH: ketoacyl synthase III
FabD: malonyl-coenzyme A transacylase
CoA-SH: coenzyme A
ACP: acyl carrier protein
MOPS: 3-morpholinopropane-1-sulfonic acid
EDTA: ethylenediaminetetraacetic acid
BME: 2-mercaptoethanol
DTT: dithiothreitol
TCA: trichloroacetic acid
CPM: 7-diethylamino-3-(4-maleimidophenyl)-4-methylcoumarin
LB: Luria-Bertani
CV: column volume
